# Tuft cells are required for a rhinovirus-induced asthma phenotype in immature mice

**DOI:** 10.1172/jci.insight.166136

**Published:** 2024-01-23

**Authors:** Yiran Li, Mingyuan Han, Shilpi Singh, Haley A. Breckenridge, Jordan E. Kreger, Claudia C. Stroupe, Daniel A. Sawicky, Shiuhyang Kuo, Adam M. Goldsmith, Fang Ke, Anukul T. Shenoy, J. Kelley Bentley, Ichiro Matsumoto, Marc B. Hershenson

**Affiliations:** 1 Department of Pediatrics,; 2 Department of Microbiology and Immunology, and; 3 Department of Internal Medicine, University of Michigan Medical School, Ann Arbor, Michigan, USA.; 4 Monell Chemical Senses Center, Philadelphia, Pennsylvania, USA.; 5 Department of Molecular and Integrative Physiology, University of Michigan Medical School, Ann Arbor, Michigan, USA.

**Keywords:** Pulmonology, Asthma, Innate immunity, Mouse models

## Abstract

Infection of immature mice with rhinovirus (RV) induces an asthma-like phenotype consisting of type 2 inflammation, mucous metaplasia, eosinophilic inflammation, and airway hyperresponsiveness that is dependent on IL-25 and type 2 innate lymphoid cells (ILC2s). Doublecortin-like kinase 1–positive (DCLK1^+^) tuft cells are a major source of IL-25. We sought to determine the requirement of tuft cells for the RV-induced asthma phenotype in wild-type mice and mice deficient in Pou2f3, a transcription factor required for tuft cell development. C57BL/6J mice infected with RV-A1B on day 6 of life and RV-A2 on day 13 of life showed increased DCLK1^+^ tuft cells in the large airways. Compared with wild-type mice, RV-infected *Pou2f3^–/–^* mice showed reductions in IL-25 mRNA and protein expression, ILC2 expansion, type 2 cytokine expression, mucous metaplasia, lung eosinophils, and airway methacholine responsiveness. We conclude that airway tuft cells are required for the asthma phenotype observed in immature mice undergoing repeated RV infections. Furthermore, RV-induced tuft cell development provides a mechanism by which early-life viral infections could potentiate type 2 inflammatory responses to future infections.

## Abstract 2

<p>In immature mice, early-life rhinovirus infection increases the development of airway tuft cells, providing a mechanism for viral-induced asthma development.</p>

## Introduction

Tuft cells of the respiratory and gastrointestinal tract are characterized by the presence of a tuft of blunt, squat microvilli (~120–140/cell) on the cell surface (1). Tuft cells are chemosensory cells that express elements of the taste transduction system, including bitter taste receptors (T2Rs), sweet and umami taste receptors (T1Rs), Gnat3 (also known as α-gustducin), and transient receptor potential cation channel subfamily M member 5 (TRPM5) (2), a calcium-activated nonselective cation channel. Tuft cells also express doublecortin-like kinase 1 (DCLK1) (3), a serine/threonine protein kinase involved in several different cellular processes, including binding of microtubules and regulation of microtubule polymerization, neuronal migration, retrograde transport, neuronal apoptosis, and neurogenesis. It has been demonstrated that Pou2f3, a POU homeodomain transcription factor, is expressed in TRPM5-expressing chemosensory cells and is necessary for their generation (4–6).

Tuft cells in the sinonasal and lung epithelia displaying taste cell–like molecular features are also termed solitary chemosensory cells, or SCCs (7). Stimulation of T2Rs in SCCs has been shown to regulate respiratory rate (2, 7), acetylcholine release (8), and a calcium wave that propagates through gap junctions to the surrounding respiratory epithelial cells, stimulating secretion of antimicrobial peptides (9). DCLK1^+^TRPM5^+^ SCCs develop in the distal lung after severe influenza injury (10–12).

Tuft cells of the respiratory and gastrointestinal tract have also been implicated in type 2 immune responses. Gastrointestinal DCLK1^+^ tuft cells constitutively express IL-25, and its expression is further increased after helminth infection, leading to IL-13 secretion by type 2 innate lymphoid cells (ILC2s) (13–15). The innate cytokines IL-25, IL-33, and thymic stromal lymphopoietin (TSLP) have additive effects on ILC2 function and cellular responsiveness to each other via upregulation of their cellular receptors (16, 17). In the airways, single-cell RNA sequencing (scRNA-Seq) has demonstrated IL-25 expression in mouse tracheal tuft cells (18, 19), and IL-25 reporter mice show that tracheal tuft cells are a primary source of IL-25 (13, 20). Aeroallergens induce DCLK1^+^IL-25^+^ tracheal tuft cell expansion in mice (20, 21) and DCLK1^+^ tuft cells produce IL-25 in patients with chronic rhinosinusitis with nasal polyps (22).

We have shown that rhinovirus A1B (RV1B) infection of 6-day-old mice, but not mature mice, induces mucous metaplasia and airway hyperresponsiveness (23) that is associated with ILC2 expansion and dependent on IL-13, IL-25, IL-33, and TSLP (17, 24). ILC2s are required and sufficient for the asthma-like phenotype (25). Finally, we observed that RV1B infection on day 6 of life augmented the impact of a second RV infection administered 1 week later, amplifying type 2 cytokine expression, mucus production, and airway resistance in an ILC2-dependent manner (26). Since DCLK1^+^ tuft cells are a major source of IL-25 in the gut (13–15), we examined the contribution of these cells to the RV-induced asthma-like phenotype in immature mice.

## Results

### Early-life RV infection expands the number of DCLK1^+^ airway tuft cells.

**Figure 1:**
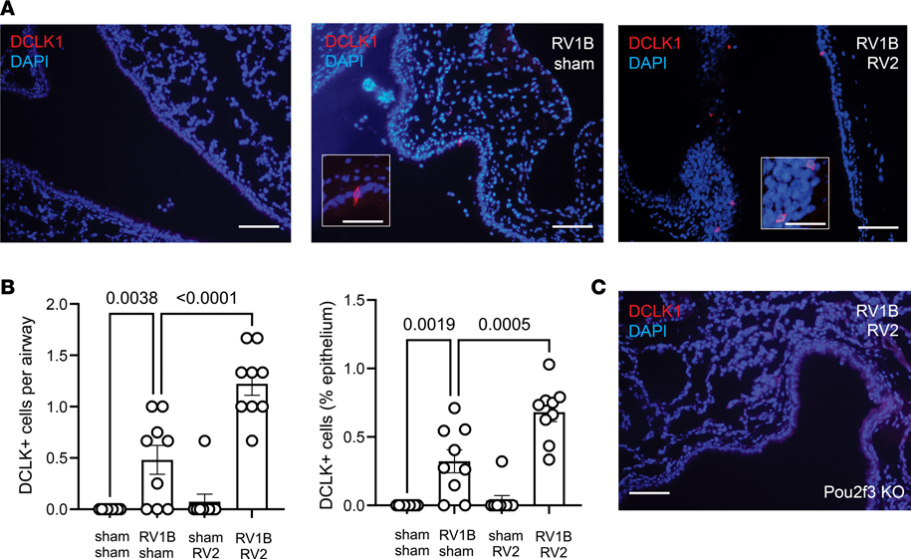
RV infection induces airway tuft cell expansion. Baby mice were inoculated with sham or RV1B on day 6 of life and sham or RV2 on day 13 of life. Lungs were harvested on day 20 and processed for immunofluorescence microscopy. (**A**) Lung sections were stained for DCLK1 (shown in red) and with DAPI (blue). Scale bars: 100 μm and 50 μm (insets). (**B**) Group mean data for the number of cells per airway (left panel) and the fraction of airway epithelium stained positively for DCLK1 (right panel). Three airways per mouse were examined and each data point represents the average for 1 mouse. Data shown are mean ± SEM (*n* = 9 mice per group from 2 different experiments) and were analyzed by 1-way ANOVA. Group differences were evaluated by Tukey’s multiple-comparison test. (**C**) Immunofluorescence images show an absence of DCLK1^+^ cells in *Pou2f3^–/–^* mice (DCLK1, red; DAPI, blue).

To determine whether early-life RV infection induces airway tuft cell expansion, wild-type C57BL/6J mice were inoculated with sham HeLa cell lysate or RV1B on day 6 of life and inoculated with sham or the heterologous RV serotype RV-A2 (RV2) on day 13 of life (26). Seven days after inoculation with sham or RV2 (day 20 of life), lungs were stained with a monoclonal antibody against DCLK1, a marker of murine tuft cells (3). Lungs from sham-infected mice showed an absence of DCLK1^+^ cells. The number and percentage of airway DCLK1^+^ cells significantly increased in mice with RV1B on day 6 of life (Figure 1, A and B). DCLK1^+^ cells were found in intrapulmonary large airways with pseudostratified or cuboidal epithelium. There was no change in the number of tuft cells following RV2 infection on day 13 of life. However, infection of mice with RV2 one week after the previous RV1B infection further increased the number and percentage of airway DCLK1^+^ cells compared with RV1B alone (Figure 1, A and B). Finally, RV-infected *Pou2f3^–/–^* mice showed an absence of DCLK^+^ cells, as expected (Figure 1C).

**Figure 2:**
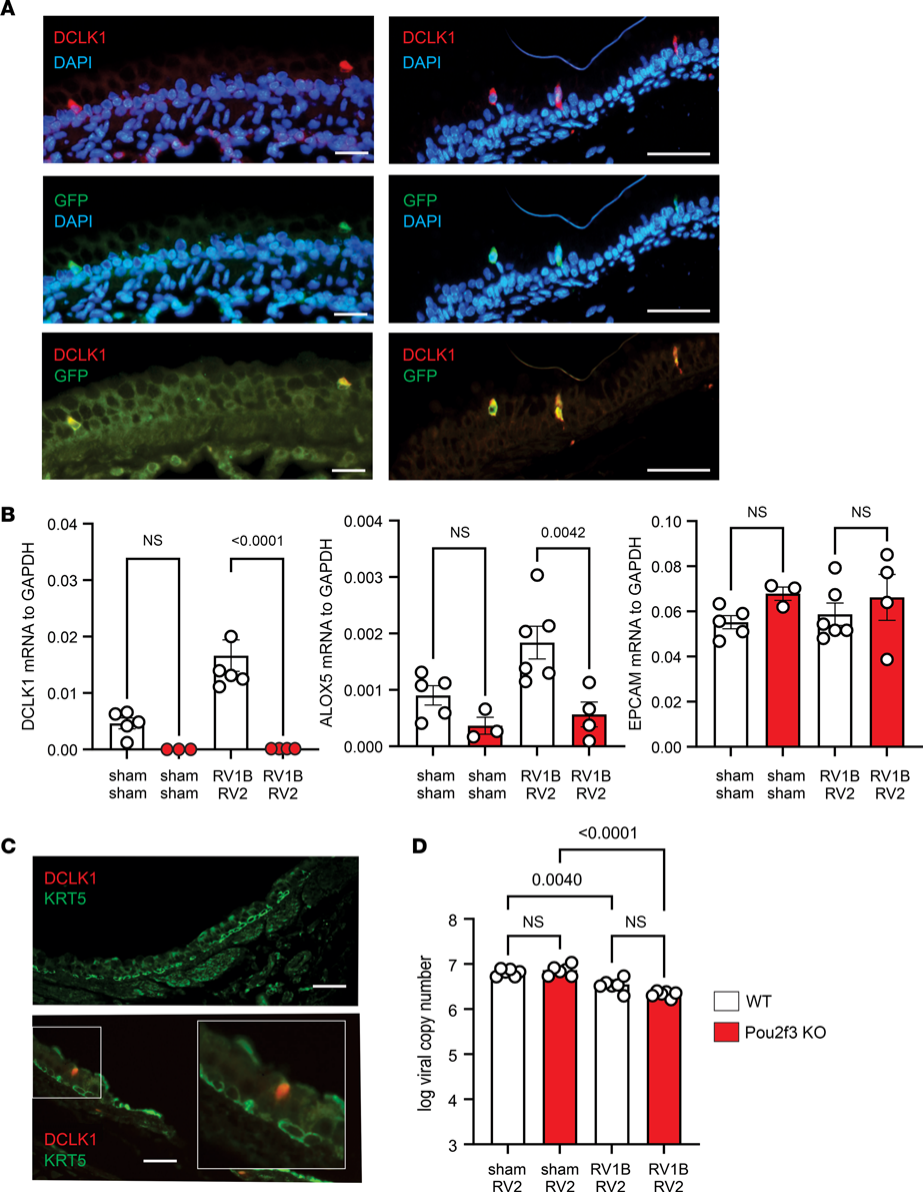
DCLK1 and GFP colocalize in the airways of RV-infected ChAT-eGFP mice. (**A**) ChAT-eGFP mice were inoculated with RV1B on day 6 of life and RV2 on day 13 of life. Lungs were harvested on day 20. Lungs were stained with anti-DCLK1 (red) and anti-GFP (green). There was colocalization of DCLK1 and eGFP (green). Scale bars: 100 μm. (**B**) Lungs from RV1B- and RV2-infected C57BL/6J and *Pou2f3^–/–^* mice were harvested on day 20 and DCLK1, arachidonate 5-lipoxygenase (ALOX5), and epithelial cell adhesion molecule (EPCAM) mRNA measured by qPCR. (**C**) Lungs and trachea from RV1B- and RV2-infected C57BL/6J mice were stained for DCLK1 (red) and Krt5 (green). Scale bars: 100 μm. (**D**) Lung viral copy number in C57BL/6J and *Pou2f3^–/–^* mice (*n* = 6 from 2 different experiments, mean ± SEM). Data were analyzed by 1-way ANOVA. Group differences were evaluated by Tukey’s multiple-comparison test.

To confirm the identity of DCLK1^+^ tuft cells, we infected immature ChAT-eGFP mice with RV1B and RV2. Choline acetyltransferase expression is part of a transcriptional signature shared by all murine tuft cells (27). We found colocalization of DCLK1 and eGFP, confirming that DLCK1^+^ cells were indeed tuft cells (Figure 2A). Also, compared with wild-type mice, lung mRNA expression of tuft cell markers *Dclk1* and *Alox5* was decreased in double-infected *Pou2f3^–/–^* mice (Figure 2B).

Krt5^+^ distal airway stem cells are essential for lung regeneration after influenza infection (28). We found Krt5^+^ cells in the trachea and rarely in the cartilaginous airways of the lung (Figure 2C). However, we were only able to find one example of Krt5^+^ cells neighboring a DCLK1^+^ tuft cell (in the trachea). Finally, we measured RV copy number 1 day after secondary infection with sham or RV2 (Figure 2D). *Pou2f3^–/–^* mice showed no changes in viral copies compared with wild-type mice.

### Pou2f3^–/–^ mice show reduced innate cytokine expression after heterologous RV infection.

**Figure 3:**
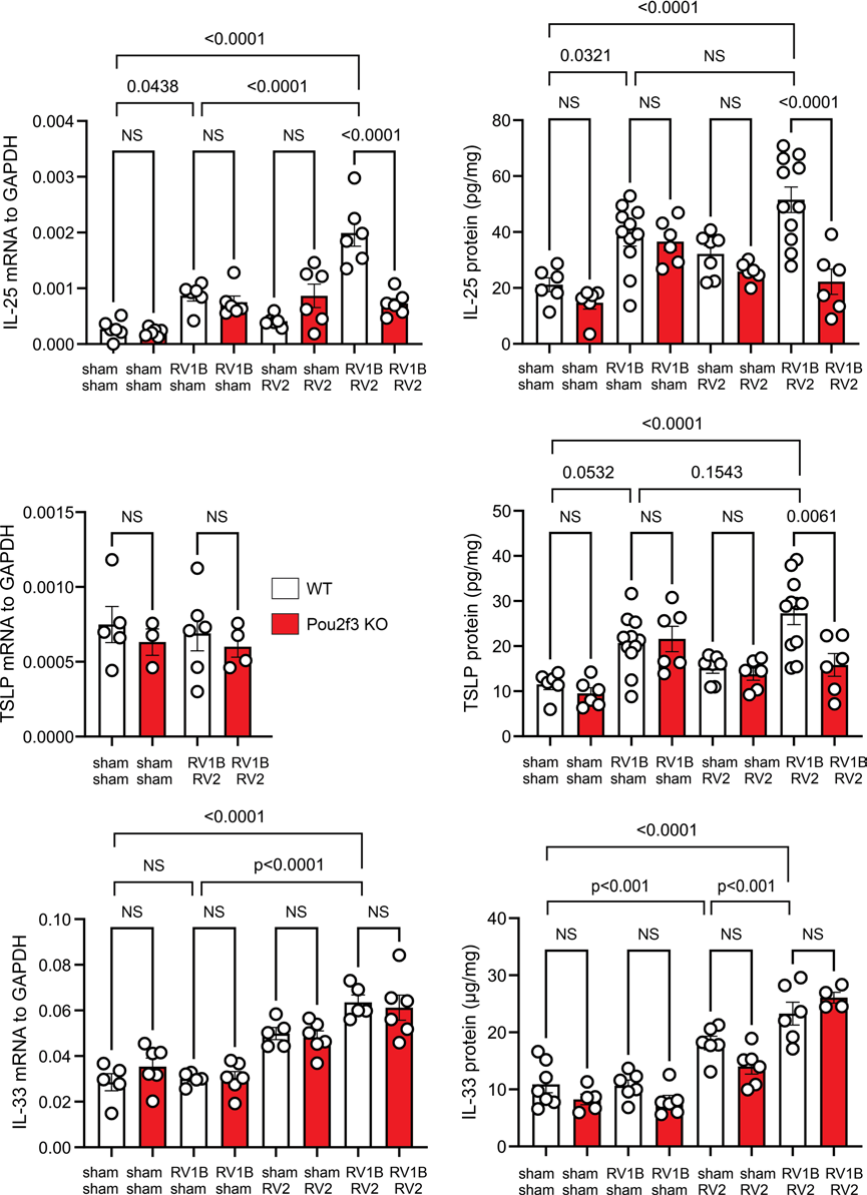
Pou2f3 deficiency blocks expression of innate cytokines in mice undergoing heterologous RV infection. Wild-type and *Pou2f3^–/–^* mice were inoculated with sham or RV1B on day 6 of life and with sham or RV2 on day 13 of life. Lungs from sham- plus sham-infected, sham- plus RV1B-infected, sham- plus RV2-infected, and RV1B- plus RV2-infected mice were harvested on day 20 for IL-25 and TSLP mRNA and ELISA and on day 14 for IL-33 mRNA and ELISA. Data shown are mean ± SEM (*n* = 6–11 per group from 2 different experiments) and were analyzed by 1-way ANOVA. Group differences were evaluated by Tukey’s multiple-comparison test.

Pou2f3, a POU homeodomain transcription factor, is required for tuft cell development (4–6). Mice with Pou2f3 deficiency show an absence of TRPM5- and DCLK1-expressing brush cells in the trachea and digestive tracts (6). We have shown that RV1B infection of 6-day-old mice induces mucous metaplasia and airway hyperresponsiveness that is associated with ILC2 expansion and dependent on IL-13, IL-25, IL-33, and TSLP (17, 24). Since the tuft cell is a major cellular source of IL-25 in the gut (13–15) and trachea (13, 18–20), we employed mice with Pou2f3 deficiency to examine the role of airway tuft cells in the innate cytokine response to heterologous RV infections. Based on previous studies showing differential kinetics for IL-25, TSLP, and IL-33 expression (17), lung mRNA and protein were harvested at day 20 of life (7 days after the last treatment) for IL-25 and TSLP and on day 14 (1 day after the last treatment) for IL-33. Mice undergoing RV1B infection on day 6 of life and sham infection on day 13 showed significantly increased mRNA and protein expression of IL-25 on day 20 of life compared with sham-infected mice (Figure 3). Heterologous infection with RV2 on day 13 of life further increased IL-25 expression. Compared with wild-type mice, double-infected *Pou2f3^–/–^* mice showed reduced mRNA and protein expression of IL-25 on day 20 of life, suggesting that tuft cells are required for the IL-25 response to heterologous RV infections. As previously shown (17), TSLP mRNA expression was not increased by viral infection. However, heterologous infection with 2 RV serotypes induced TSLP protein (Figure 3). Compared with wild-type mice, double-infected *Pou2f3^–/–^* mice showed reduced TSLP expression on day 20 of life, suggesting that tuft cells are also required for RV-induced TSLP protein production. However, the Pou2f3 deficiency did not attenuate IL-25 or TSLP expression in mice treated with a single RV infection. Finally, double infection with 2 RV serotypes significantly increased both IL-33 mRNA and protein expression. IL-33, which is not produced by tuft cells, was unaffected by the Pou2f3 deficiency (Figure 3).

### Pou2f3^–/–^ mice show reduced lung ILC2s.

**Figure 4:**
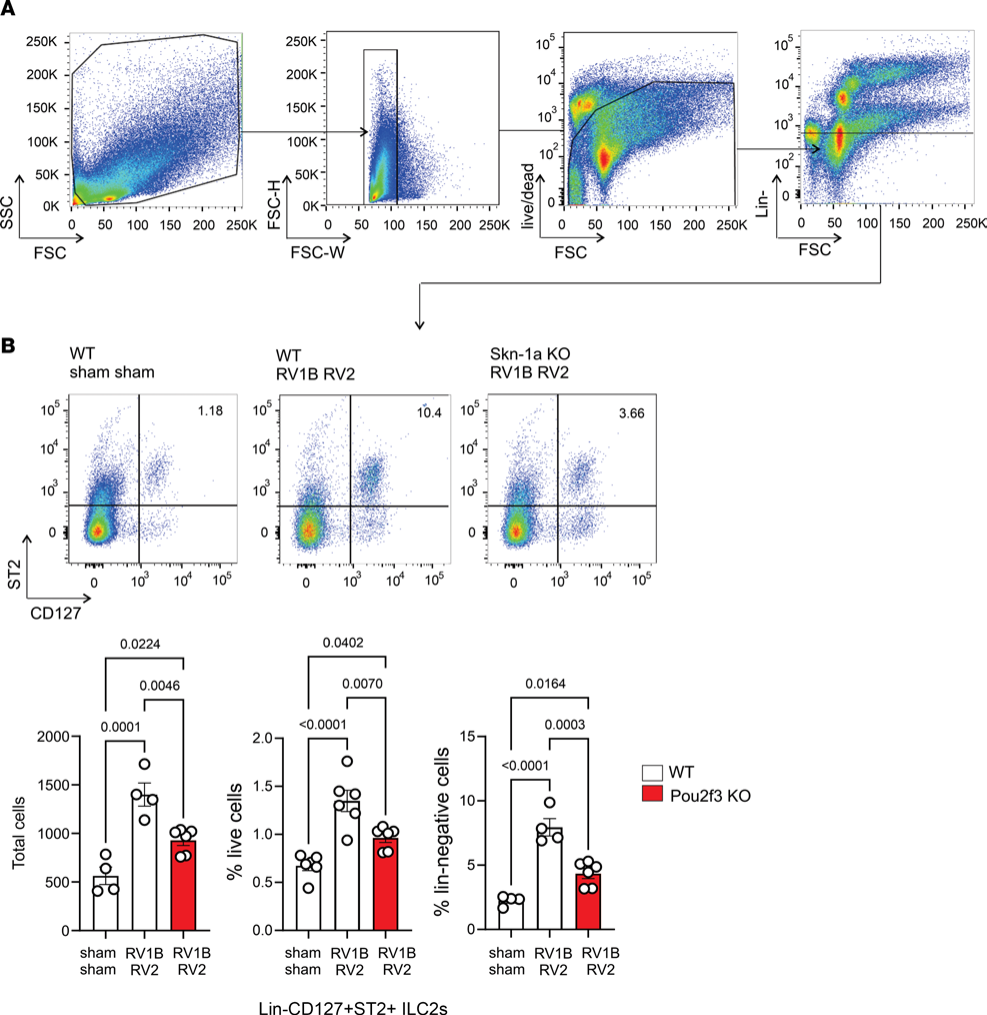
Pou2f3 deficiency attenuates lung ILC2 expansion in 6-day-old mice following heterologous RV infection. Wild-type and *Pou2f3^–/–^* mice were inoculated with sham or RV1B on day 6 of life and sham or RV2 on day 13 of life. On day 20, lungs were harvested, digested with Liberase, collagenase XI, hyaluronidase 1a, and DNase I, and stained with Pacific blue (for dead cells), lineage antibody cocktail, anti-CD127, and anti-ST2. Cells were washed, fixed, and processed for flow cytometry. (**A** and **B**) Flow cytometric analysis of live Lineage^–^CD127^+^ST2^+^ ILC2s from sham + sham and RV1B + RV2 groups. Group mean data for ILC2s are shown (*n* = 4–6 from 2 different experiments, mean ± SEM). Data were analyzed by 1-way ANOVA. Group differences were evaluated by Tukey’s multiple-comparison test.

In our model, IL-25, IL-33, and TSLP cooperate in RV-induced ILC2 expansion and mucous metaplasia, each having additive effects on ILC2 function and augmenting cellular responsiveness to each other via upregulation of their cellular receptors (17). We therefore examined the requirement of Pou2f3 for ILC2 expansion 1 week after heterologous RV infections. Consistent with our previous results (26), infection with RV1B and RV2 increased the number of Lineage^–^ST2^+^CD127^+^ cells by flow cytometry (Figure 4, A and B). ILC2 number was significantly reduced, though not abolished, in *Pou2f3^–/–^* mice following heterologous infection (Figure 4B).

### Pou2f3^–/–^ mice show reduced type 2 inflammation after double RV infection.

**Figure 5:**
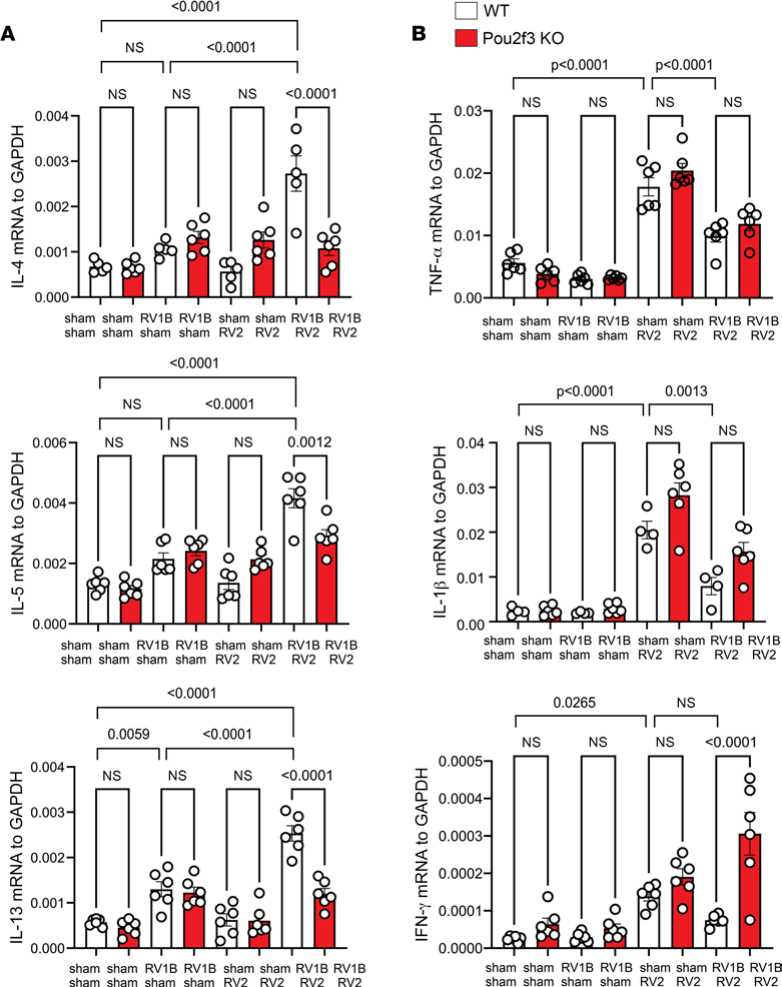
Pou2f3 deficiency blocks enhanced expression of type 2 inflammation in mice undergoing heterologous RV infection. Mice were inoculated with sham or RV1B on day 6 of life and sham or RV2 on day 13 of life. (**A**) Lungs were harvested on day 20 and processed for mRNA expression of IL-4, IL-5, and IL-13. Data shown are mean ± SEM (*n* = 6 per group from 2 different experiments) and were analyzed by 1-way ANOVA. (**B**) mRNA expression of the proinflammatory cytokines IL-1β, TNF-α, and IFN-γ. Lungs were harvested for qPCR on day 14 of life, 1 day after secondary sham or RV2 infection (*n* = 6 from 2 different experiments, mean ± SEM). Data were analyzed by 1-way ANOVA. Group differences were evaluated by Tukey’s multiple-comparison test.

To determine the downstream effects of airway tuft cell deficiency, we measured cytokine mRNA expression in wild-type C57BL/6J and tuft cell–deficient *Pou2f3^–/–^* mice. Lungs were harvested for type 2 cytokine mRNA and protein on day 20 of life and harvested for proinflammatory cytokine mRNA and protein on day 14 of life. In contrast with mice infected with a single RV serotype, heterologous infection with RV2 on day 13 of life induced exaggerated type 2 cytokine mRNA expression. Double-infected *Pou2f3^–/–^* mice showed significantly reduced mRNA expression of IL-4, IL-5, and IL-13 (Figure 5A), demonstrating that tuft cells are required for the type 2 cytokine response to heterologous RV infection.

In contrast with IL-4, IL-5, and IL-13, mRNA expression of IL-1β and TNF-α, both considered type 1 cytokines, decreased after infection with RV2, suggesting that RV infection on day 6 of life skewed the response to subsequent heterologous RV infection toward a type 2 phenotype (Figure 5B). Double-infected *Pou2f3^–/–^* mice did not show a change in TNF-α or IL-1β mRNA cytokine expression. Unexpectedly, *Pou2f3^–/–^* mice infected with both serotypes showed increased IFN-γ mRNA expression compared with mice infected with RV1B alone.

### Effects of Pou2f3 deficiency on the asthma phenotype.

**Figure 6:**
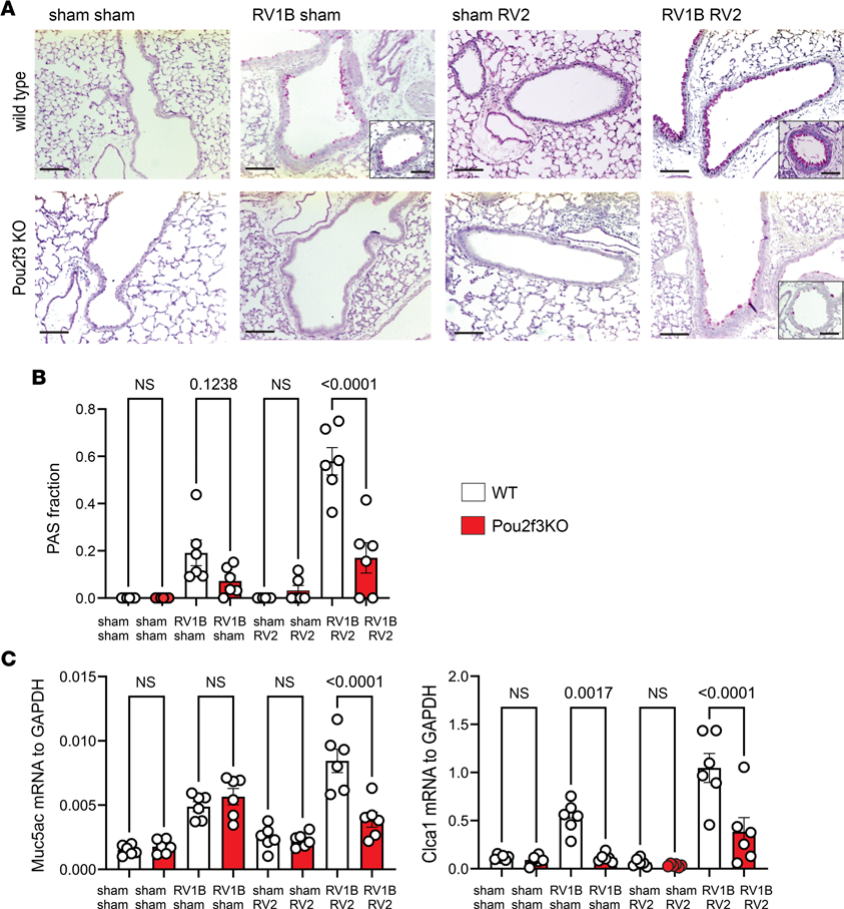
Pou2f3 deficiency blocks exaggerated mucus metaplasia in mice undergoing heterologous RV infection. Mice were inoculated with sham or RV1B on day 6 of life and sham or RV2 on day 13 of life. Lungs were harvested on day 20 and processed for histology and measurement of mRNA expression. (**A** and **B**) Lung sections were stained with PAS. (**A**) Large airways are shown, except for insets which show small airways. (**B**) The fraction of epithelium stained positively for PAS was quantified using NIH ImageJ software. Two to 3 airways were examined for each mouse. Scale bars: 200 μm and 100 μm (insets). (**C**) mRNA expression of the mucus-associated genes *Muc5ac* and *Clca1*. Data shown are mean ± SEM (*n* = 6 per group from 2 different experiments) and were analyzed by 1-way ANOVA. Group differences were evaluated by Tukey’s multiple-comparison test.

**Figure 7:**
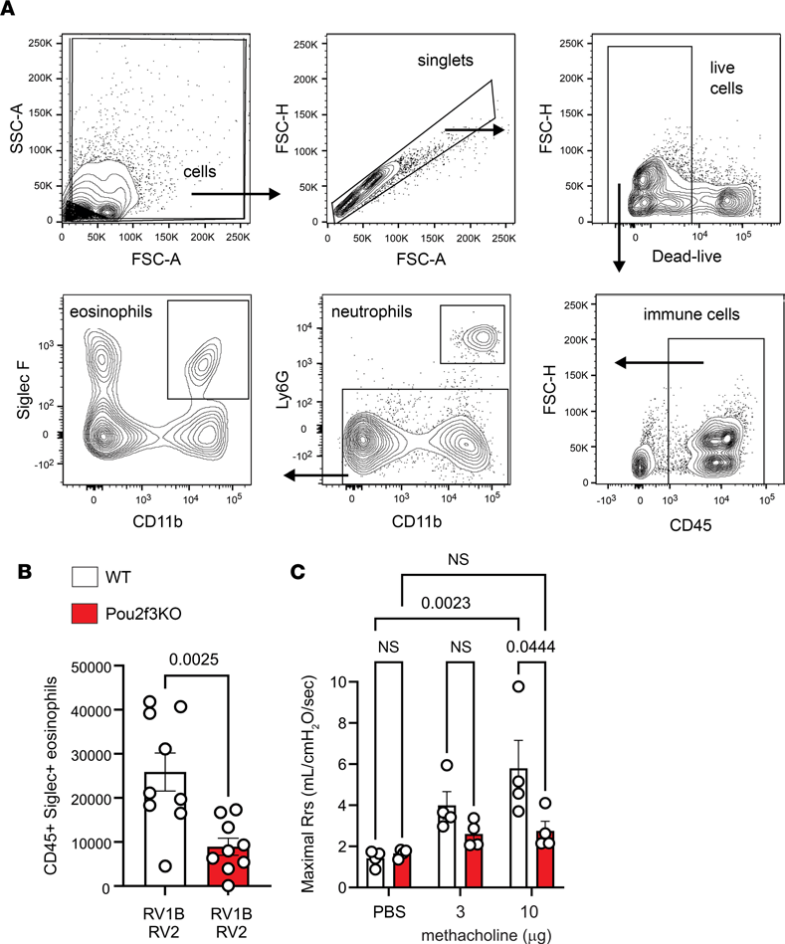
Pou2f3 deficiency blocks the asthma phenotype in RV-infected immature mice. Wild-type and *Pou2f3^–/–^* mice were infected with RV1B on day 6 of life and sham or RV2 on day 13 of life. (**A** and **B**) To identify eosinophils, lung cells were stained for CD45, Siglec-F, and CD11b. Data shown are mean ± SEM (*n* = 9 per group from 2 different experiments) and were analyzed by 1-way ANOVA. (**C**) Airway resistance was measured in anesthetized, intubated, and ventilated mice before and after administration of methacholine. Data shown are mean ± SEM (*n* = 4 per group from a single experiment) and were analyzed by 2-way ANOVA. Group differences were evaluated by Tukey’s multiple-comparison test.

In contrast with a single infection with RV1B, heterologous RV infections increased the number of periodic acid–Schiff–positive (PAS-positive) cells (Figure 6, A and B). A similar pattern was observed for the mRNA expression of Muc5ac and Gob5 (Figure 6C). Since PAS detects mucosubstances such as glycoproteins, glycolipids, and mucins, together these data indicate that heterologous RV infection induces mucous metaplasia. In contrast, *Pou2f3^–/–^* mice lacking tuft cells exhibited attenuated PAS staining. Next, we measured lung eosinophils by flow cytometry. Compared with wild-type RV1B- and RV2-infected mice, double-infected *Pou2f3^–/–^* mice showed reduced lung eosinophils (Figure 7, A and B). Finally, we measured changes in airway resistance in RV-infected mice after injection of methacholine into the retro-orbital venous sinus (Figure 7C). *Pou2f3^–/–^* mice showed a significantly lower response to methacholine than wild-type mice.

## Discussion

Tuft cells of the lower airway epithelium are chemosensory cells that express TRPM5, a calcium-activated cation channel of the bitter taste transduction system (2), and DCLK1, a serine/threonine protein kinase involved in the regulation of microtubule polymerization. In addition to a chemosensory function, tuft cells also play a role in inflammation. In mice, DCLK1^+^ chemosensory cells develop in the distal lung after severe influenza injury (10–12). Gastrointestinal DCLK1^+^ tuft cells constitutively express IL-25, and its expression is further increased after helminth infection, leading to IL-13 secretion by ILC2s (13–15). In the respiratory system, *Alternaria* and house dust mite exposure induce DCLK1^+^IL-25^+^ airway tuft cell expansion (20). Tracheal tuft cells are a primary source of IL-25 in mice (13, 18–20), and tuft cells produce IL-25 in human patients with chronic rhinosinusitis with nasal polyps (22).

Wheezing-associated respiratory infections with RV are associated with asthma development later in life (29–31). On average, children experience 4 acute respiratory infections the first year of life, the majority of which are RV (32). To determine whether RV infection early in life could promote development of an asthma phenotype, we infected 6-day-old mice with RV1B. We found that early-life RV infection increased lung ILC2s, mucous metaplasia, and airway hyperresponsiveness (23, 24). We also observed that RV1B infection on day 6 of life augmented the impact of a second RV infection administered 1 week later, amplifying type 2 cytokine expression, mucus production, and airway resistance in an ILC2-dependent manner (26). We found that epithelial cell–derived innate cytokines cooperate to promote the RV-induced phenotype, each having additive effects on ILC2 function and augmenting cellular responsiveness to each other via upregulation of their cellular receptors (17). We therefore investigated whether infection with RV in early life modulates tuft cell development. We found that early-life RV infection induced the appearance of DCLK1^+^ tuft cells and that heterologous infection with both RV1B and RV2 caused a further increase in DCLK1^+^ cells. We also determined the requirement of tuft cells for the RV-induced asthma phenotype in wild-type mice and mice deficient in Pou2f3, a transcription factor required for tuft cell development. *Pou2f3^–/–^* mice undergoing heterologous RV infections showed significant reductions in IL-25 and TSLP protein expression, ILC2 expansion, type 2 cytokine expression, mucous metaplasia, lung eosinophils, and airway methacholine responsiveness. We conclude that airway tuft cells, by virtue of their production of IL-25, are required for the exaggerated asthma phenotype observed in immature mice undergoing repeated RV infections. As far as we aware, this is the first report demonstrating the requirement of tuft cells for a virus-induced asthma phenotype. Tuft cells have previously been shown to be required for allergic airway disease in *Alternaria*-exposed mice (21, 33).

We did not determine the mechanism by which viral infection increases tuft cell development. Epithelial damage itself could elicit a remodeling program that leads to tuft cell development. As noted above, DCLK1^+^ chemosensory cells develop in the distal lung after severe influenza injury (10, 11). After influenza infection, DCLK1^+^TRPM5^+^ SCCs coexpress p63 and arise near Krt5^+^ cells (10), suggesting a common origin with p63^+^Krt5^+^ distal airway epithelial cells implicated in lung regeneration (34). However, after RV infection, DCLK1^+^ tuft cells were found primarily in the proximal intrapulmonary airways and were not associated with Krt5^+^ cells. This is consistent with the minimal damage to the airway epithelium after RV infection (35). Other mechanisms promoting tuft cell development are possible; in the intestine, tuft cell hyperplasia following parasitic infection is dependent on IL-13–producing ILC2s (13). Finally, aeroallergen-induced airway tuft cell expansion in adult mice is dependent on cysteinyl leukotrienes (20).

While Pou2f3-deficient mice lacking tuft cells showed an attenuated response to double RV infection, the limited response to a single infection on day 6 of life was generally maintained. Specifically, IL-25 and TSLP production in RV1B-induced Pou2f3-deficient mice was not decreased, suggesting that tuft cells are not the only cellular source of these cytokines in 6-day-old mice. Alveolar macrophages, eosinophils, and other immune cells may also produce IL-25 (36, 37). Another possibility is that tuft cells do not require Pou2f3 during early postnatal development.

We would like to comment on the small number of tuft cells we found in the airways of immature mice infected with RV. Airways from uninfected mice showed no DCLK^+^ cells. After heterologous RV infection, the number of tuft cells constituted approximately 1% of the airway epithelium. The paucity of DCLK1^+^ cells is consistent with previous studies employing scRNA-Seq showing that tuft cells constitute a rare population of chemosensory cells in the airways (18). Previous studies have found few (<1%) (12) or no tuft cells (10, 33, 38) beyond the trachea in naive mice. On the other hand, recent studies in influenza-infected (10) and allergen-challenged mice (21, 33), as well as human patients with chronic rhinosinusitis, show them to be more numerous (22). Factors influencing the number of tuft cells identified may include age, species, airway generation, intensity of the provoking stimulus, length of time after the provoking stimulus, and method of identification, for example scRNA-Seq versus immunostaining. Also, it is conceivable that we missed a subset of DCLK1-negative tuft cells (21).

We conclude that airway IL-25–producing tuft cells are required for the exaggerated asthma phenotype observed in immature mice undergoing repeated RV infections. Furthermore, RV infection increases airway tuft cell development, providing a potential mechanism by which early-life viral infections could potentiate type 2 inflammatory responses to future infections, skewing the immune response toward an asthma phenotype.

## Methods

### Animals.

C57BL/6J mice and B6.Cg-Tg(RP23-268L19-EGFP)2Mik/J (ChAT-eGFP mice) were purchased from The Jackson Laboratory. To elucidate the function of tuft cells in our model, we employed *Pou2f3^–/–^* mice, as described elsewhere (4). Mice were bred in house in a pathogen-free facility within the Unit for Laboratory Animal Medicine at the University of Michigan.

### Generation of RV1B and RV2.

RV1B and RV2 (ATCC), minor-group viruses that infect mouse cells (39), were partially purified from infected HeLa cell lysates by means of ultrafiltration with a 100-kDa cutoff filter and titered by using a plaque assay as described previously (40, 41). Intact virions do not go through the filter and are concentrated. Similarly concentrated and purified HeLa cell lysates were used for sham infection.

### RV infections.

Mice were inoculated with 15 μL of HeLa cell lysate, 1.5 × 10^6^ plaque-forming units (PFU) of RV1B or 1.5 × 10^6^ PFU of RV2 through the intranasal route under Forane anesthesia. Mice were treated as follows: (a) day 6 sham and day 13 sham, (b) day 6 RV1B and day 13 sham, (c) day 6 sham and day 13 RV2, and (d) day 6 RV1B and day 13 RV2.

### Real-time quantitative PCR.

Lung RNA was extracted with TRIzol (Invitrogen) and isolated using an RNeasy kit (Qiagen). cDNA was synthesized from 2 μg of RNA using a High Capacity cDNA Synthesis kit (Applied Biosystems) and subjected to quantitative real-time PCR (qPCR) using specific primers (Table 1) for mRNA. The level of gene expression for each sample was normalized to GAPDH. To quantify viral copy number, qPCR for positive-strand viral RNA was conducted using RV-specific primers and probes (forward primer: 5′-GTGAAGAGCCSCRTGTGCT-3′; reverse primer: 5′-GCTSCAGGGTTAAGGTTAGCC-3′; probe: 5′-FAM-TGAGTCCTCCGGCCCCTGAATG-TAMRA-3′).

### Histology and immunofluorescence.

Lungs were fixed with 10% formaldehyde overnight and paraffin embedded. Blocks were sectioned at 500-μm intervals at a thickness of 5 μm, and each section was deparaffinized, hydrated, and stained. To visualize mucus, sections were stained with PAS (Sigma-Aldrich). PAS staining in the airway epithelium was quantified by NIH ImageJ software. Six separate mouse lungs of either wild-type or *Pou2f3^–/–^* mice from each of the 4 conditions were processed for sectioning. Two to 3 separate airways of similar size from each lung were chosen for analysis. PAS abundance was calculated as the fraction of PAS-positive epithelium compared with the total basement membrane length. Images were visualized using an Olympus IX71 microscope with appropriate filters. For quantification of tuft cells, lung sections were stained with 4′,6-diamidino-2-phenylindole (DAPI) and anti–mouse DCLK1 antibody (catalog 62257, clone D2U3L, Cell Signaling Technology) followed by Alexa Fluor 555 anti–rabbit IgG secondary antibody (catalog A-11036, Invitrogen). Nine separate mouse lungs of either wild-type or *Pou2f3^–/–^* mice from each of the 4 conditions were processed for sectioning. Three separate airways from each lung were chosen for analysis, and the data are presented as an average for each lung. Images were visualized using a Nikon E800 microscope with appropriate filters. Finally, additional tissue sections were stained with anti-Krt5 (catalog 905501, clone Poly19055, BioLegend) or anti-eGFP (catalog A10262, Invitrogen) antibody followed by Alexa Fluor anti–chicken IgY (catalog A-11039, Invitrogen).

### Flow cytometric analysis.

Lungs were perfused with PBS containing 0.5 mM EDTA and minced and digested with Liberase (100 μg/mL ; Roche), collagenase XI (250 μg/mL; Sigma-Aldrich), hyaluronidase 1a (1 mg/mL; Sigma-Aldrich), and DNase I (200 μg/mL; Sigma-Aldrich) for 1 hour at room temperature. Cells were filtered and washed with RBC lysis buffer (BD Biosciences). Nonspecific binding was blocked with 1% fetal bovine serum with 1% LPS-free bovine serum albumin in DMEM, and 5 μg rat anti–mouse CD16/32 (catalog 101302, clone 93, BioLegend) was added. To identify ILC2s, cells were stained with FITC-conjugated antibodies for lineage markers CD3ε, B220/CD45R, Ter-119, Gr-1/Ly-6G/Ly-6C, CD11b (all catalog 78022, BioLegend), CD11c (catalog 117306, clone N418, BioLegend), TCRβ (catalog 109206, clone H57-597, BioLegend), F4/80 (catalog 123108, clone BM8, BioLegend), FcεRIα (catalog 134306, clone MAR-1, BioLegend), anti-CD127–allophycocyanin (anti-CD127–APC) (catalog 121122, clone SB/199, eBioscience), and anti-ST2–phycoerythrin-cyanine 7 (anti-ST2–PE/Cy7) (catalog 145316, clone DIH9, BioLegend), as described previously (42). To identify eosinophils, cells were stained with anti-CD45–eFluor 450 (catalog 48-0451-82, clone 30-F11, Invitrogen), anti–Siglec-F–APC-Cy7 (catalog 565527, clone E50-2440, BD Biosciences), and anti-CD11b–APC (catalog 101212, clone M1/70, BioLegend) and negatively gated for anti-CD11c–PE/Cy7 (catalog 558079, clone HL3, BD Biosciences) and anti-Ly6G–PE (catalog 551461, clone 1A8, BD Biosciences).

### Measurement of IL-25, IL-33, and TSLP protein levels.

Lung IL-25, IL-33, and TSLP were measured by ELISA (Thermo Fisher Scientific). ELISA data were analyzed by BioTek Gen5 software. Total lung protein concentration was measured by BCA protein assay (Thermo Fisher Scientific).

### Measurement of airway responsiveness.

Thirty-day-old mice were anesthetized, intubated, and ventilated with a Buxco FinePointe System (Data Sciences International). Methacholine was administered via retroorbital injection (43).

### Statistics.

All data are represented as mean ± SEM. For studies of airway responsiveness, statistical significance was assessed by 2-way ANOVA. For all other experiments, statistical significance was assessed by 1-way ANOVA. Group differences were evaluated by Tukey’s multiple-comparison test. In each case, means of each treatment group were compared with the mean of every other group, but for clarity only selected comparisons are shown.

### Study approval.

All animal usage was approved by the University of Michigan Institutional Animal Care and Use Committee (protocol number PRO00010065) and followed guidelines set forth in the NIH *Guide for the Care and Use of Laboratory Animals* (National Academies Press, 2011).

### Data availability.

Underlying data are available in the supplemental Supporting Data Values XLS file.

## Author contributions

YL helped design the research, performed experiments, analyzed data, interpreted results of the experiments, prepared figures, drafted the manuscript, and approved the final version of the manuscript. With MH’s permission, YL was assigned first position because he reliably identified tuft cells by DCLK1 immunofluorescence and verified using ChAT-eGFP mice. MH helped design the research, performed experiments, analyzed data, interpreted results of experiments, prepared figures, drafted the manuscript, and approved the final version of the manuscript. SS, HAB, JEK, CCS, DAS, SK, AMG, and FK performed experiments. ATS designed research, analyzed data, and prepared figures. JKB edited and revised the manuscript. IM conceived and designed the research, edited and revised the manuscript, and approved the final version of the manuscript. MBH conceived and designed research, analyzed data, prepared figures, edited and revised the manuscript, and approved the final version of manuscript.

## Supplementary Material

Supporting data values

## Figures and Tables

**Table 1:**
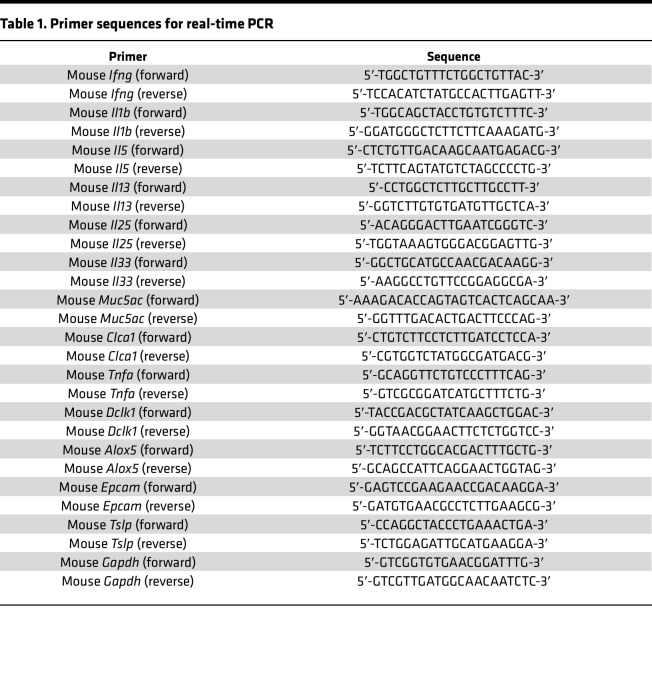
Primer sequences for real-time PCR

## Version 1 12/07/2023

In-Press Preview

## Version 2 01/23/2024

Electronic publication
